# Preoperative prediction of hepatocellular carcinoma with portal vein tumor thrombus based on conventional data

**DOI:** 10.18632/oncotarget.22198

**Published:** 2017-10-31

**Authors:** Pengpeng Zhu, Yan Liao, Jiyuan Fan, Xin Li, Lili Su, Jun Li, Shengguang Yuan, Junxiong Yu, Weijia Liao

**Affiliations:** ^1^ Laboratory of Hepatobiliary and Pancreatic Surgery, Affiliated Hospital of Guilin Medical University, Guilin, Guangxi, P.R. China; ^2^ Disease Prevention and Control Center of Guilin, Guilin, Guangxi, P.R. China; ^3^ Department of Pathology and Pathophysiology, Xiangya hospital, Central South University, Changsha, P.R. China; ^4^ Department of Clinical Laboratory, Nanxishan Hospital of Guangxi Zhuang Autonomous Region, Guilin, Guangxi, P.R. China; ^5^ Department of Hepatobiliary and Pancreatic Surgery, Affiliated Hospital of Guilin Medical University, Guilin, Guangxi, P.R. China; ^6^ Department of Anesthesiology, The Second Affiliated Hospital of Guilin Medical University, Guilin, Guangxi, P.R. China

**Keywords:** hepatocellular carcinoma, portal vein tumor thrombosis, prediction, clinical index, survival

## Abstract

Hepatocellular carcinoma (HCC) has a high predilection with portal vein tumor thrombosis (PVTT). However, part of the PVTT type can be found only under the microscopy, which was namely as type I_0_. The objective of this study was to establish a simple and inexpensive non-invasive model to predict the presentation of PVTT at HCC patients. A total of 815 HCC patients were retrospectively evaluated and randomly assigned into 2 groups: the training group (n = 408) and validation group (n = 407). A new index model, namely WγAL, was built to predict the presence of PVTT in the training subjects and was further validated in the validation subjects. At the optimal cutoff of 8.90, WγAL identified patients with a hazard ratio (HR) of 7.139 for the presence of PVTT. The area under receiver operating characteristic (AUROC) of WγAL was 0.795 (sensitivity: 71.9%; specificity: 78.6%) for differentiation between PVTT and non-PVTT patients in the training group. The AUROC of WγAL in differentiating patients with PVTT type I_0_ from non-PVTT patients was 0.748 (sensitivity: 72.1%; specificity: 68.4%) with an HR of 5.355. In addition, WγAL > 8.90 was significantly associated with large tumors, multiple tumor numbers, TNM stage III-IV, metastasis, and overall survival in HCC patients. The novel predictive model represents a simple and inexpensive model that can identify the presence of PVTT in HCC patients with a high degree of accuracy, with important clinical significance in the future therapeutic management of HCC patients.

## INTRODUCTION

Hepatocellular carcinoma (HCC) is one of the aggressive malignancies with a high prevalence worldwide that has been ranked as the sixth most common malignant diseases and the third most frequent cause of death from cancer [1, 2]. Recent studies have indicated that the overall clinical survival of HCC remains extremely poor because of the high incidence of recurrence after surgical resection, or intra-hepatic metastases that come from invasion of the portal vein branches [3, 4]. Portal vein tumor thrombosis (PVTT), a main route for intra-hepatic or distant metastases of HCC cells in human HCC patients, is usually associated with an unsatisfactory prognosis [5-7]. The median survival time is only 2 to 4 months compared with 10 to 24 months in those without PVTT [8]. In addition, there may be 50% to 80% of HCC patients with portal or hepatic vein invasion [9]; however, among HCC individuals with a tumor diameter less than 2 cm, 40.5% have a very high likelihood of developing venous invasion [10]. Additionally, the presence of PVTT in HCC directly determines the appropriate treatment modality [11].

PVTT occurs in a substantial section of HCC patients at the time of diagnosis and has an evident adverse effect on prognosis due largely to the high risk of wide tumor transmission and increased pressure of the portal vein, resulting in acute variceal hemorrhage, and lowered portal flow causing intractable ascites, jaundice, hepatic encephalopathy and failure. The current approach to detect PVTT is magnetic resonance imaging, ultrasonography and surgical exploration. However, part of PVTT was a vexing topic because it was visible only by microscopic examination of the surgical specimen. While some special effort has been made to predict PVTT, there has been no robust clinical application caused by many restrictive factors. Accordingly, a model that can not only precisely identify venous metastasis but also be widely used in the clinic is a highly desirable goal for HCC patients.

The aim of this study was to create a simple formula using age and readily available laboratory parameters at low cost and with high precision to predict PVTT in a cohort of HCC patients and to guide treatments effectively in clinical practice.

## RESULTS

### Patient cohorts

A total of 815 patients with HCC were included. Among the entire cohort, the mean age was 49.91 ± 11.27 years. Most of the patients were male (86.75%) and were predominantly hepatitis B carriers (83.68%) and cirrhotic patients (92.52%). Among the 815 patients, 234 (28.71%) patients had PVTT: type I_0_ (90, 11.04%), type I (72, 8.83%), type II (51, 6.26%) and type III (21, 2.58%).

Among the 815 patients, 408 and 407 patients were assigned randomly to the training and validation cohort, respectively. The demographic, clinical, and laboratory data of the training and validation cohorts are listed in Table 1. Overall, there were no significant differences in the baseline characteristics between HCC patients in these two groups. Patients in the two groups had similar age distribution, WBC and lymphocyte count, and serum γ-GT levels.

**Table 1:** Clinical and biochemical data of examined patients

Parameter	Training cohort (n = 408)	Validation cohort (n = 407)	*P* value
Age, years	50.16±11.86	49.65±10.66	0.520
Gender: female/male (n)	54/354	54/353	0.989
Family history: yes/no (n)	59/349	56/351	0.774
Drinking: yes/no (n)	206/202	180/227	0.073
Smoking: yes/no (n)	197/211	216/191	0.172
HBsAg: negative/positive (n)	74/334	59/348	0.160
HCVAb: negative/positive (n)	398/10	395/12	0.661
Cirrhosis: yes/no (n)	368/40	386/21	0.012
WγLA value	10.83±12.95	10.76±12.90	0.935
WBC, ×10^9^/L	6.29±2.32	6.43±2.18	0.387
LYMPH, ×10^9^/L	1.62±0.60	1.67±0.61	0.186
Platelets, ×10^9^/L	174.93±82.55	184.32±79.01	0.097
Albumin, g/L	40.11±4.65	38.78±4.79	0.103
Globulin, g/L	29.95±5.72	31.56±6.07	0.076
TBIL, μmol/L	17.42±26.62	6.46±22.71	0.582
DBIL, μmol/L	7.09±17.87	7.75±19.76	0.616
ALT, U/L	47.71±43.65	47.49±50.12	0.948
AST, U/L	53.27±45.39	54.34±47.89	0.743
AFP, ng/ml: median, range	121.50 (0.31-242000.00)	116.00 (0.23-328030.00)	0.405
γ-GT, U/L: median, range	88.50 (15.00-802.00)	82.28 (10.00-777.10)	0.622

^*^Data presented as mean ± SD or proportions.

N, number of patients; HBsAg, hepatitis B surface antigen; HCVAb, hepatitis C virus antibody; WγLA, γ-GT [U/L] × (WBC [10^9^/L]) / (age [years] × LYMPH [10^9^/L]); WBC, white blood cell; LYMPH, lymphocyte count; TBIL, total bilirubin; DBIL, direct bilirubin; ALT, alanine aminotransferase; AST, aspartate aminotransferase; AFP, alpha-fetoprotein; γ-GT, γ-glutamyl transpeptidase.

### Development of a novel model to predict PVTT

Univariate logistic analysis showed that gender, age, median size, tumor number, TNM stage, distant metastasis, serum ALT, AST, γ-GT, WBC and lymphocyte count were a risk factor for HCC with PVTT (data not shown). Multivariable analysis using backward stepwise procedures identified age, serum γ-GT, WBC count and lymphocyte count, *etc.* as independent predictors of PVTT. A novel index called the WγAL was calculated according to the following formula: γ-GT [U/L] × (WBC [10^9^/L]) / (age [years] × LYMPH [10^9^/L]). The mean value of WγAL was 10.83±12.95 in the training group and 10.76±12.90 in the validation cohort (Table 1).

### Accuracy of the WγAL index model in predicting the presence of PVTT

**Figure 1:**
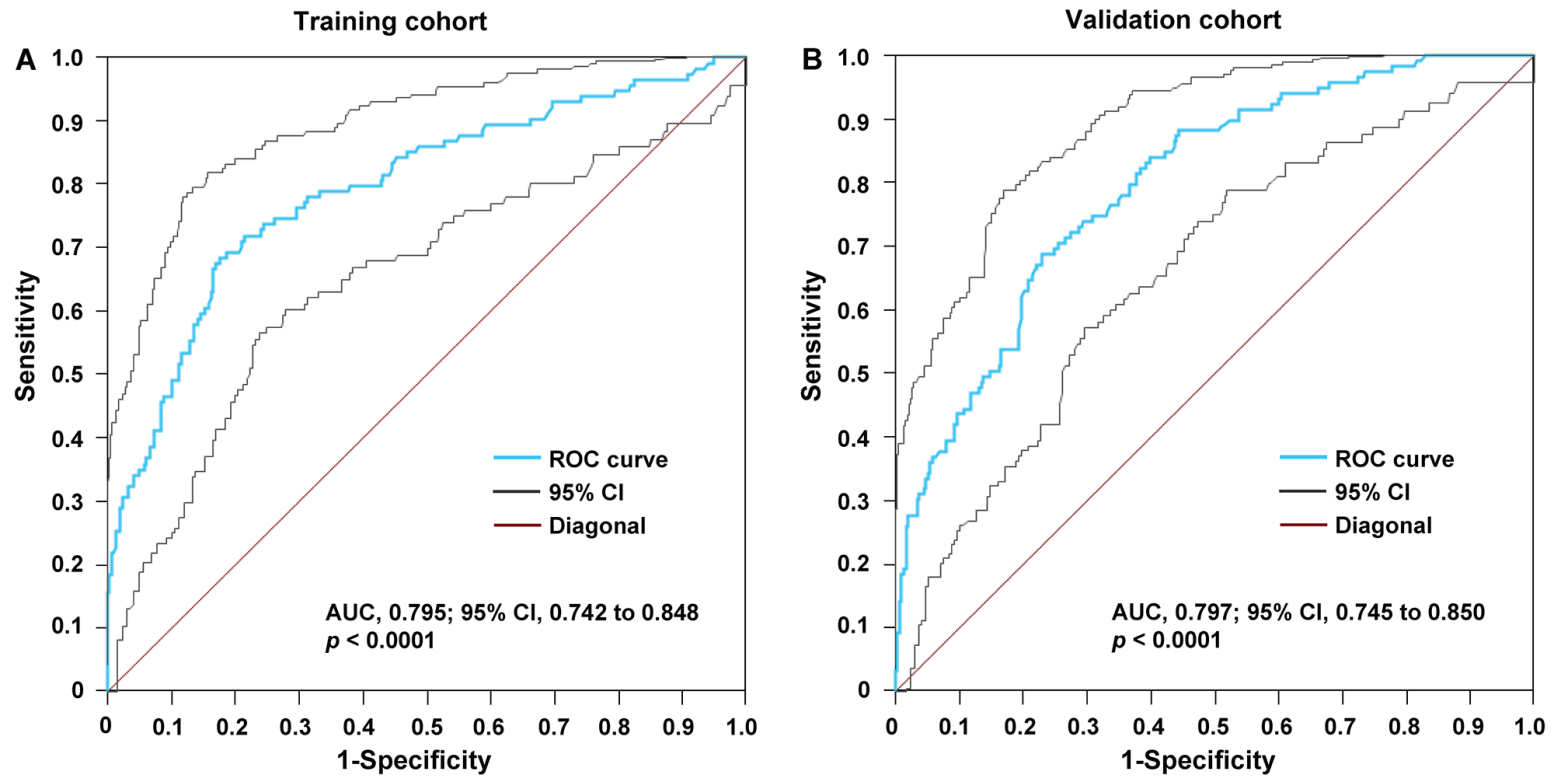
ROC plot was used for the prediction of the presence of PVTT using the WγAL index in the **(A)** training and **(B)** validation groups. The WγAL had an AUROC of 0.795 for the training group and an AUROC of 0.797 for the validation set.

The ROC curves for WγAL differentiating the presence of PVTT from non-PVTT patients are shown in Figure 1. The AUROC of WγAL was 0.795 (95% CI, 0.742 to 0.848) for the differentiation between PVTT and non-PVTT patients in the training group (*P* < 0.0001), and the optimal cutoff value was 8.90. The WγAL > 8.90 predicted the presence of PVTT with an HR of 8.022. At a cutoff of ≤ 8.90 in the training group, the negative predictive value to exclude the presence of PVTT was 87.8% with a sensitivity of 71.9%, whereas a WγAL > 8.90 would have a specificity of 78.6% and a positive predictive value of 56.6% for the presence of PVTT (Table 2). The model was also tested in the validation group using the threshold value identified in the training set, and the results were similar to those obtained from the training cohort (Table 2).

**Table 2:** Accuracy of the WγLA Index in predicting portal vein tumor thrombosis in the groups

Group	AUROC	Sensitivity	Specificity	NPV	PPV	HR
Training^†^	0.795	0.719	0.786	0.878	0.566	8.022
95% CI	0.742 to 0.848	0.627 to 0.799	0.734 to 0.831	0.833 to 0.915	0.481 to 0.648	5.294 to 12.041
Validation^†^	0.797	0.706	0.743	0.859	0.532	6.917
95% CI	0.745 to 0.850	0.615 to 0.786	0.689 to 0.793	0.810 to 0.901	0.451 to 0.613	4.308 to 11.105
Non-PVTT + I_0_^‡^	0.748	0.721	0.684	0.891	0.335	5.355
95% CI	0.696 to 0.763	0.618 to 0.816	0.644 to 0.715	0.850 to 0.923	0.273 to 0.429	3.636 to 8.082

^†^ Cut-off values for training and validation groups were 8.90. ^‡^Cut-off values for Non-PVTT + I_0_ group was 8.72.

Non-PVTT, Hepatocellular carcinoma (HCC) without portal vein tumor thrombosis (PVTT); I_0,_ HCC with PVTT type I_0;_ AUROC, area under the receiver operating characteristics curve; HR, hazard ratio; CI, confidence interval; NPV, negative predictive value; PPV, positive predictive value.

As it is impossible to detect PVTT type I_0_ (tumor thrombi formation found under microscopy) before surgery, the γWAL was applied to an independent set of patients without PVTT and with PVTT type I_0_. For predicting presence of PVTT type I_0_, the optimal cutoff value of WγAL was 8.72. And WγAL > 8.72 predicted the HCC with PVTT type I_0_ with an HR of 5.355. The AUROC was 0.748 (95% CI, 0.696 to 0.763) for WγAL to predict patients with PVTT type I_0_ versus those without PVTT, accompanied with a sensitivity of 72.1%, specificity of 68.4%, PPV of 33.5%, NPV of 89.1% (Table 2).

### Association of the WγAL levels with the PVTT type and TNM stage

**Figure 2:**
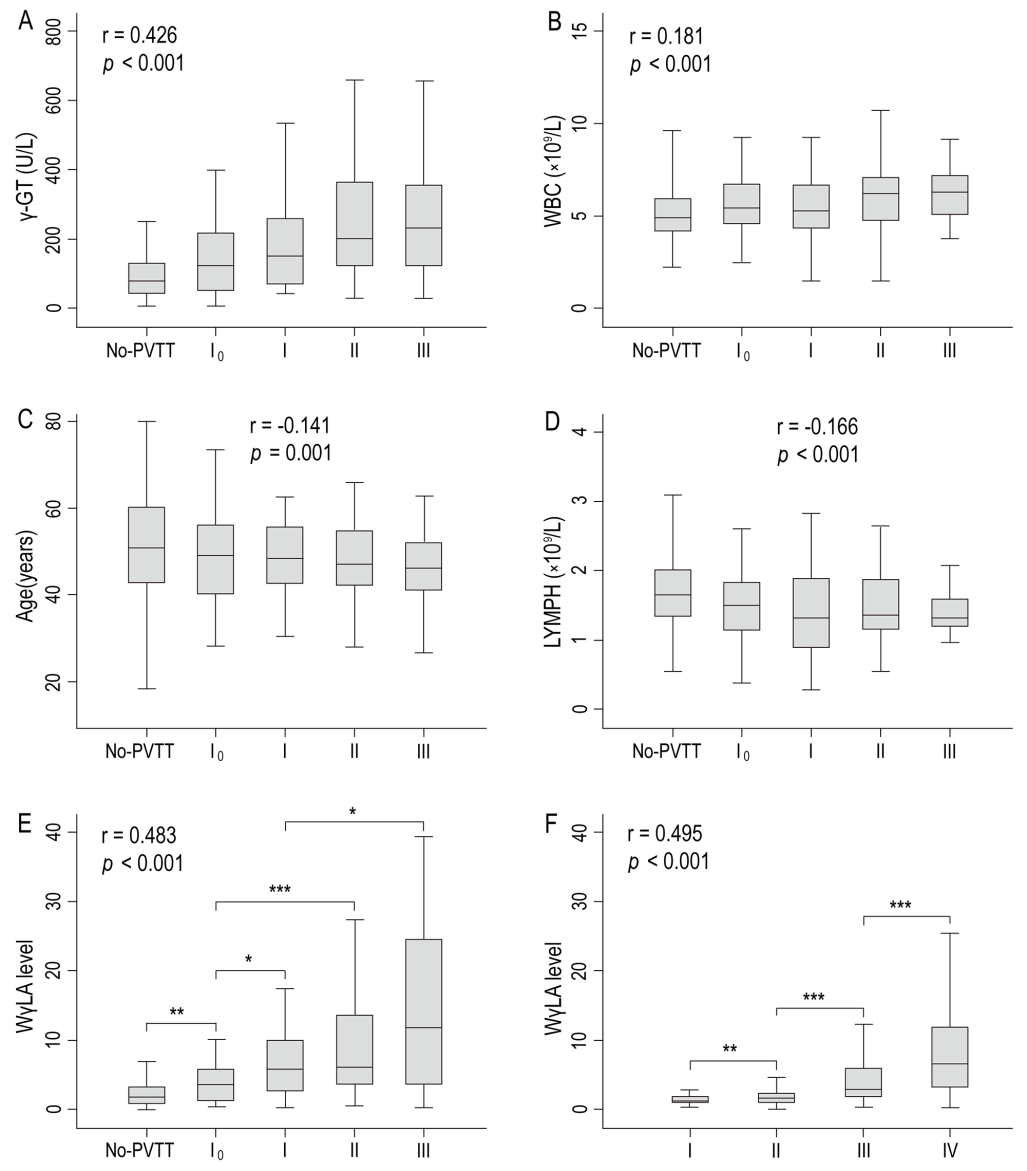
Box plots of **(A)** serum γ-GT level, **(B)** WBC count, **(C)** age, **(D)** LYMPH count, and **(E)** WγAL index level according to the PVTT type, and **(F)** WγAL index level in relation to TNM stage in the training cohorts (^*^, *P* **< 0.** 05; ^**^, *P* < 0.01; ^***^, *P* < 0.001).

In the training cohort, 239 patients had no PVTT, and 115 patients had PVTT: type I_0_ (n = 46), type I (n = 34), type II (n = 23), type III (n = 12). Box plots of serum γ-GT level, WBC count, age, lymphocyte count, and WγAL level in relation to the PVTT type are presented in Figure 2A-2E. While serum γ-GT level and WBC count had positive correlations with PVTT type (Spearman’s correlation coefficient r = 0.426, *P* < 0.001 and r = 0.181, *P* < 0.001, respectively), age and lymphocyte count were negatively correlated (r =-0.141, *P* = 0.001 and r =-0.166, *P* < 0.001, respectively). Of note, WγAL level was correlated significantly with the PVTT type, with a higher correlation coefficient than serum γ-GT level, WBC count, age, or lymphocyte count alone (r = 0.481, *P* < 0.001). Moreover, the severity of tumor-node-metastasis (TNM) stage [stage I (n = 48), stage II (n = 128), stage III (n = 147), stage IV (n = 85)] was also correlated significantly with a gradual increase in the WγAL level (r = 0.495, *P* < 0.001, Figure 2F).

In the validation cohort, 288 patients had no PVTT, and 119 patients had PVTT: type I_0_ (n = 44), type I (n = 38), type II (n = 28), type III (n = 9). The WγAL level was also correlated significantly with the PVTT type (r = 0.450, *P* < 0.001), with a higher correlation coefficient than serum γ-GT level (r = 0.430, *P* < 0.001), WBC count (r = 0.251, *P* < 0.001), age (r = -0.138, *P* = 0.011), or lymphocyte count (r = -0.189, *P* < 0.001) alone (Supplementary Figure 1A-1E, supporting information). In addition, the increase of WγAL level was correlated significantly with stage of TNM [stage I (n = 54), stage II (n = 136), stage III (n = 135), stage IV (n = 82)] (r = 0.446, *P* < 0.001, Supplementary Figure 1F, supporting information). The current findings suggested that the elevation of WγAL level may contribute to the progression of HCC by promoting invasion and metastasis.

### Correlation of WγAL with clinicopathologic features in the training group

To examine whether the WγAL index was associated with the clinicopathological parameters of HCC patients in the training group, we first classified the patients into low WγAL subjects (≤ 8.90) and high WγAL subjects (> 8.90) according to the results of the ROC curve. The following factors were identified to be positively associated with the WγAL index in the training group: gender (*P* = 0.045), age (*P* = 0.016), median size (*P* < 0.001), tumor number (*P* = 0.031), TNM stage (*P* < 0.001), the presence of PVTT (*P* < 0.001), metastasis (*P* < 0.001), serum AFP levels (*P* < 0.001), serum ALT levels (*P* < 0.001), serum AST levels (*P* < 0.001), serum γ-GT level (*P* < 0.001), WBC count (*P* < 0.001), and lymphocyte count (*P* < 0.001) (Table 3), suggesting that the increase of WγAL is positively correlated with liver inflammation and damage and further affects the malignant biological behaviour of HCC.

**Table 3:** Correlation between the clinicopathologic variables and WγLA index in training and validation cohorts

	Training cohort WγLA level			Validation cohort WγLA level		
Clinical character	≤ 8.90	> 8.90	*P* value	≤ 8.90	> 8.90	*P* value
	n = 268	n = 140		n = 252	n = 155	
Age, years	51.17±11.57	48.21±12.19	0.016	51.57±10.64	46.52±9.97	< 0.001
Male sex, n (%)	226 (84.32)	128 (91.43)	0.045	210 (83.33)	143 (92.26)	0.010
HBsAg positive, n (%)	224 (83.58)	110 (78.57)	0.212	213 (84.52)	135 (87.09)	0.474
Median size, cm	7.02±4.42	11.51±5.83	< 0.001	6.589±3.94	10.58±6.06	< 0.001
With cirrhosis, n (%)	242 (90.30)	126 (90.00)	0.923	238 (94.44)	148 (95.48)	0.645
Multiple tumors, n (%)	86 (32.09)	60 (42.86)	0.031	75 (29.76)	66 (42.58)	0.008
TNM stage, I/II/ III/IV	47/107/87/27	1/21/60/58	< 0.001	50/113/60/29	4/23/75/53	< 0.001
With PVTT, n (%)	35 (13.06)	80 (57.14)	< 0.001	36 (14.28)	83 (53.54)	< 0.001
Metastasis, n (%)	20 (7.46)	31 (22.14)	< 0.001	18 (7.14)	37 (23.87)	< 0.001
AFP, log_10_ ng/ml	2.04±1.14	2.40±1.17	< 0.001	1.89±1.22	2.52±1.14	< 0.001
ALT, U/L	40.96±34.65	60.61±54.82	< 0.001	36.89±35.67	64.72±63.77	< 0.001
AST, U/L	41.92±29.48	74.98±60.33	< 0.001	43.55±43.46	71.87±49.68	< 0.001
γ-GT, log_10_ U/L	1.70±0.27	2.27±0.27	< 0.001	1.68±0.25	2.26±0.24	< 0.001
WBC, ×10^9^/L	5.81±1.97	7.23±2.64	< 0.001	5.97±1.75	7.19±2.59	< 0.001
LYMPH, ×10^9^/L	1.73±0.63	1.39±0.48	< 0.001	1.74±0.57	1.58±0.65	0.010

N, number of patients; HBsAg, hepatitis B surface antigen; TNM, tumor-node-metastasis; PVTT, portal vein tumor thrombosis; Metastasis, distant metastasis or lymph node metastasis; AFP, alpha-fetoprotein; ALT, alanine aminotransferase; AST, aspartate aminotransferase; γ-GT, γ-glutamyl transpeptidase; WBC, white blood cell; LYMPH, lymphocyte count.

### Correlation of WγAL with clinicopathologic features in validation cohort

We further performed a correlation analysis between the WγAL index and clinicopathological variables in the validation HCC cohort. Similar to the findings in the training group, there were significant correlations between the WγAL index and thirteen parameters, including gender (*P* = 0.010), age (*P* < 0.001), median size (*P* < 0.001), tumor number (*P* = 0.008), TNM stage (*P* < 0.001), the presence of PVTT (*P* < 0.001), metastasis (*P* < 0.001), serum AFP levels (*P* < 0.001), serum ALT levels (*P* < 0.001), serum AST levels (*P* < 0.001), serum γ-GT level (*P* < 0.001), WBC count (*P* < 0.001), and lymphocyte count (*P* = 0.010) (Table 3).

### Correlation of the WγAL level and different PVTT type with the prognosis of patients in the training cohort and validation cohort

**Figure 3:**
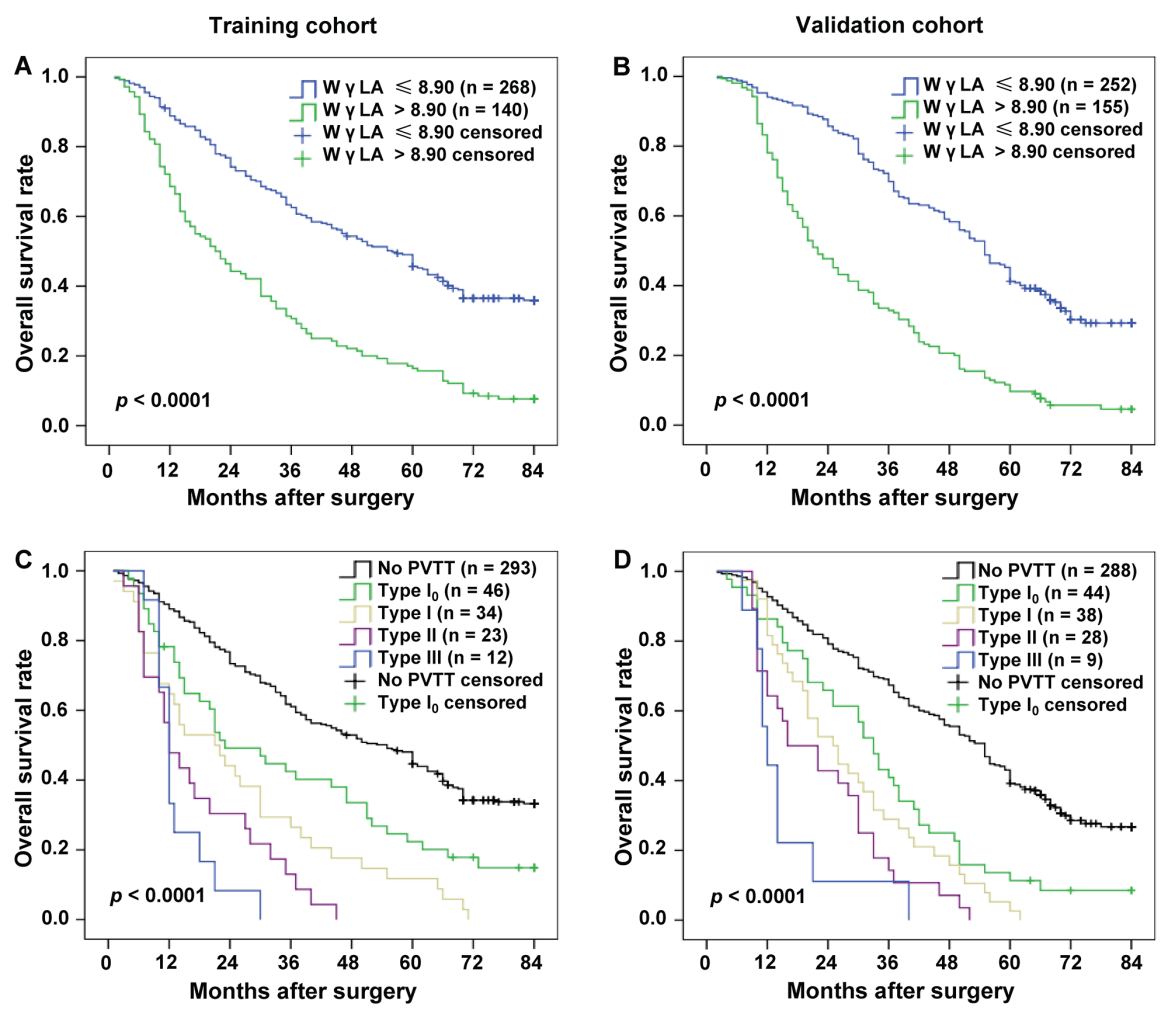
Kaplan–Meier estimated survival curves by the WγAL level and PVTT type in the training and validation cohort WγAL > 8.90 had a shorter overall survival in training **(A)** and validation **(B)** cohorts, and the PVTT type was also significantly associated with overall survival in both training **(C)** and validation **(D)** cohorts (all *P* < 0.0001).

In the training cohort, Kaplan-Meier survival analysis showed that the mean overall survival was 30.04 months (95% CI, 25.99-34.10) in high WγAL level subjects (> 8.90) and 52.33 months (95% CI, 48.86-55.79) in low WγAL level subjects (≤ 8.90) (*P* < 0.0001, Figure 3A). And overall survival significantly decreased with worsening PVTT type (Figure 3C). Median survival was, in fact, 51.36 (95% CI, 48.07-54.65) months in patients without PVTT, 36.39 (95% CI, 28.42-44.35) months in type I_0_, 26.85 (95% CI, 19.21-34.50) months in type I, 17.78 (95% CI, 12.62-22.95) months in type II, and 13.92 (95% CI, 10.36-17.47) months in type III (*P* < 0.0001, Figure 3C). The prognostic values of the WγAL level and different PVTT type were further confirmed in the validation cohort (Figure 3B and 3D). Compared with the high WγAL level subjects (> 8.90), the low WγAL level subjects (≤ 8.90) had longer mean overall survival (30.16 months, 95% CI, 26.85-33.46 and 54.18 months, 95% CI, 51.09-57.28, respectively; *P* < 0.0001). Furthermore, the PVTT type was also significantly associated with the mean overall survival (no PVTT: 51.82 months, 95% CI, 48.80-54.84; type I_0_: 35.10 months, 95% CI, 28.83-41.38; type I: 28.84 months, 95% CI, 23.61-34.07; type II: 22.75 months, 95% CI, 17.83-27.67; type III: 14.67 months, 95% CI, 9.73-19.60; *P* < 0.0001). These results suggest that WγAL level and PVTT type were closely related to the prognosis of HCC patients.

## DISCUSSION

Clinically, tumor thrombi invaded into the portal vein can be noted in most HCC patients when diagnosed, especially for late-stage HCC, which is associated significantly with a poorer prognosis [12]. It has been reported in the literature that PVTT is the major factor influencing the median OS duration of postoperative HCC patients [13]. In this study, we demonstrated that survival of patients after surgery progressively decreased with the increasing severity of PVTT type. The Barcelona Clinic Liver Cancer (BCLC) Staging System is the most widely embedded in HCC management guidelines, which categories HCC subjects with PVTT into BCLC stage C and recommend sorafenib as a standard therapy for those people [14, 15]. However, most Asian centres tend to be more aggressive in treatment, especially in surgery. PVTT type I or II patients with a primary tumour may be treated with surgical treatment and may obtain a radical cure, and PVTT type III patients may choose surgery, transarterial chemoembolization (TACE) or radiotherapy combined with TACE after surgical resection according to the tumor thrombus. It is obvious that the early diagnosis of PVTT patients, especially type I_0_ stage of PVTT patients, is of great significance. Increasing studies have been reported a long-term survival outcome in patients with PVTT who have undergone hepatic resection [16, 17]. The sufficient and effective preoperative identification of venous metastasis on non-invasive markers in patients with HCC is of great benefit, which may guide the timely treatment planning and successful intervention in the early development of HCC and provide a more accurate assessment of prognosis in HCC patients.

PVTT or its molecular mechanism was reportedly predicted by exome sequencing, gene expression profiling, microRNA microarray techniquesand serum biomarkers [11, 18-20]. These studies have exhibited value to predicting the occurrence of PVTT but with time-consuming, power-wasting, or inconvenient operation to some degree. As of now, concerning the precision medicine and treatment of HCC, people have endeavoured to search the “Liquid biopsies” of human peripheral blood, which play a clinical pivotal role in the early warning and prognosis evaluation of HCC [21]. However, the diagnostic and predictive abilities for HCC with PVTT are finite with a single index or individual molecules; additionally, in relative terms, a prediction model including multiple clinical factors for the prediction of PVTT is worth pursuing in HCC patients.

In the present study, we have developed a novel model using a cost-effective procedure and relatively high precision to classify HCC patients with PVTT, based on the γ-GT, WBC, age and lymphocyte status. Importantly, WγAL can predict the presence of PVTT type I_0_ in HCC patients with high discrimination ability. PVTT type I_0_ had always been undetected by preoperative imaging techniques, possibly promoting intrahepatic spreading through portal vessel cancer cell dissemination. Thus, there is an urgent need for an accurate and objective method to predict PVTT type I_0_ before surgery. Encouragingly, our results have shown that WγAL > 8.72 has an HR of 5.355 for predicting the presence of PVTT type I_0_ in HCC patients, demonstrating that this non-invasive WγAL index may be a promising marker of predicting hepatic PVTT type I_0_ in HCC. As PVTT type I_0_ is detected only by postoperative pathological microscopic examination, the significance of this new index is more reflected in the preoperative judgment of this PVTT type. And it is of great significance in assessing whether surgery should be performed, choosing appropriate resection type (anatomical or non-anatomical), or expanding the scope of liver resection to achieve the effect of radical resection under the condition of preserved liver function.

As an integrated indicator based on the γ-GT, WBC, and lymphocyte status, the predictive value of the WγAL for the incidence of PVTT might be elucidated by the function of these three types of factors. Moreover, these three factors play a different role in the development and progression of cancer. First, as one of the important serum liver enzymes, γ-GT is routinely tested in liver function tests for evaluating host liver function. Human γ-GT is responsible for the synthesis and degradation of glutathione, biotransformation, and nucleic acid metabolism [22, 23]. The striking elevation of γ-GT may act as a surrogate of oxidative stress, damaging cells and posing a hazard for humans and is significantly associated with the tumorigenesis of HCC [24, 25]. It has been reported that serum γ-GT was higher in patients with more advanced liver disease and larger tumors [26, 27]. Second, a higher count of circulating WBCs, acting as a well-standardized and available marker of systemic inflammation in routine clinical checkups, is significantly associated with an increased risk of cancer disease and mortality [28, 29]. The expression of various cytokines and inflammatory mediators may be increased in an environment rich in inflammatory cells, significantly precipitating the proliferation and metastasis of malignant cells via the inhibition of apoptosis pathways and DNA damage [30, 31]. Finally, lymphocytes play powerful roles in defending against the tumor malignancy within the tumor microenvironment [32]. Relative lymphocytopenia in an HCC host may compromise antitumor immune effects. First, CD4^+^ T lymphocytes are required to eliminate (pre) malignant tumor cells, inhibit HCC initiation, and mediate tumor regression [33, 34]. A dramatic fall in the CD4^+^ T lymphocyte level greatly influenced the anti-tumor activity that may lead to a fast progression of HCC. Second, with the support of perforin and granzyme, CD8^+^ cytotoxic T lymphocytes (CTLs) can directly contact and kill tumor cells [35]. There is persuasive evidence that the depletion of CTLs may promote the invasion of tumor cells into the surrounding tissue and metastasis to new body sites; collectively, an increased number of CTLs in HCC is closely connected to a reduced tumor diameter and longer survival times [36, 37]. Moreover, hepatic NK cells have the power to kill malignant tumor cells by releasing cytokines. Once inhibited by the increased number of peripheral Tregs, they may not successfully trigger the clearance of infected cells and malignant cells [38, 39].

Accordingly, these three factors contributed to endow HCC cells with the potential of invasion and metastasis in a different way. It can be speculated that the HCC comprise mostly tumor cells with the strong ability of metastatic and vascular invasion in the patients with elevated WγAL levels. Kaplan-Meier survival analysis showed that the survival was significantly poor in high WγAL level subjects compared with low WγAL level subjects. Meanwhile, our results indicated that the WγAL level had an upward tendency with the severity of PVTT and TNM stage, further confirming this hypothesis.

Consistent with real-life that HCC tends to occur in adolescents and young adults, our correlation analysis showed a negative relationship between the WγAL value and age. Of note, an elevated WγAL value demonstrated a positive correlation with the median tumor size, tumor number, TNM stage, distant metastasis, and serum ALT and AST levels in both the training and validation HCC groups, confirming that an increasing WγAL value may play an indispensable role not only in the malignant phenotype and metastasis of HCC but also in the damaging inflammation of the liver. There is scant evidence that tumors due to a malignant PVTT have a large trend towards larger diameters compared with those due to a benign thrombosis [40]. Clinically, PVTT is a more frequent condition in patients with the terminal stage of HCC [41]. Therefore, patients with a higher preoperative WγAL value should be closely monitored, and appropriate measures should be applied timely to prevent the growth of HCC diseases.

The insights provided in our study are limited by its retrospective nature and two-institution study design. A prospective and multi-centre study is needed to confirm the predictive effect of the WγAL index on PVTT in further research. To the best of our knowledge, this study shows for the first time that the WγAL index is useful for predicting the risk of PVTT. A significant observation that stems from our study is that the WγAL index has a higher HR for predicting type I_0_ of PVTT in HCC patients. The elevation of the WγAL value was significantly positively related to the PVTT stage, and adverse tumor biology of HCC, which may be a promising method for the preoperative prediction of PVTT in HCC during clinical practice.

## MATERIALS AND METHODS

### Patients

From February 1993 to May 2013, 815 HCC patients undergoing surgical resection were reviewed retrospectively at the Affiliated Hospital of Guilin Medical University and Nanxishan Hospital of Guangxi Zhuang Autonomous Region, Guangxi, China. All of these patients were randomly assigned into a training set (n = 408) or a validation set (n = 407). The diagnosis was according to clinical, serological, ultrasonography, computerized tomography, magnetic resonance imaging, and pathologic examination, and PVTT in all of the patients was staged into different type according to the uniform tumor thrombus type system of the Eastern Hepatobiliary Surgery Hospital of China [42]. This study was approved by the research ethics committee of the participating hospital and complied with The Declaration of Helsinki Principles. Informed consent was obtained from all patients.

All of the demographic data and laboratory investigations were recorded at the time of HCC diagnosis including age, gender, family history, drinking status, smoking status, cirrhosis, hepatitis B surface antigen, hepatitis C virus antibody, white blood cell (WBC) count, lymphocyte (LYMPH) count, platelet count, and albumin, globulin, total bilirubin, direct bilirubin, alanine aminotransferase (ALT), aspartate aminotransferase (AST), AFP, and γ-glutamyl transpeptidase (γ-GT) levels.

Patients were excluded from the analysis if they had any of the following criteria: 1) a confirmed diagnosis of cholangiocellular carcinoma or a diagnosis that ruled out HCC based on pathological detection; 2) insufficient demographic, clinical or laboratory data; 3) confirmed evidence of immunodeficiency, hematological disorders, or concomitant tuberculosis.

### Statistical analysis

The SPSS software package, version 18.0 (SPSS Inc., Chicago, IL, USA) was used for statistical analysis. Univariate and multivariate logistic analysis were used to identify predictors of PVTT. Receiver operating characteristic (ROC) curve analysis was used to detect the most appropriate cut-off value. For the evaluation of the new index to predict PVTT, the area under the ROC curve (AUROC), sensitivity, specificity, positive predictive value (PPV), negative predictive value (NPV), and hazard ratio (HR) were calculated. Overall survival was calculated using the Kaplan–Meier method and compared with the log-rank test. Continuous variables were assessed by Student’s t test or nonparametric tests. Pearson’s χ^2^ test was performed to compare the categorical variables. In all tests, *P* values were statistically significant if less than 0.05.

## SUPPLEMENTARY MATERIALS FIGURE


