# Supplementary material for: Preoperative prediction of hepatocellular carcinoma with portal vein tumor thrombus based on conventional data
Source: Oncotarget. 2017 Oct 31;8(61):104227–37. doi: 10.18632/oncotarget.22198 (PMC5732801; doi:10.18632/oncotarget.22198)
Supplement: Supplementary file 1 [file oncotarget-08-104227-s001.pdf]

Preoperative prediction of hepatocellular carcinoma with portal vein tumor thrombus based on conventional data

SUPPLEMENTARY MATERIALS

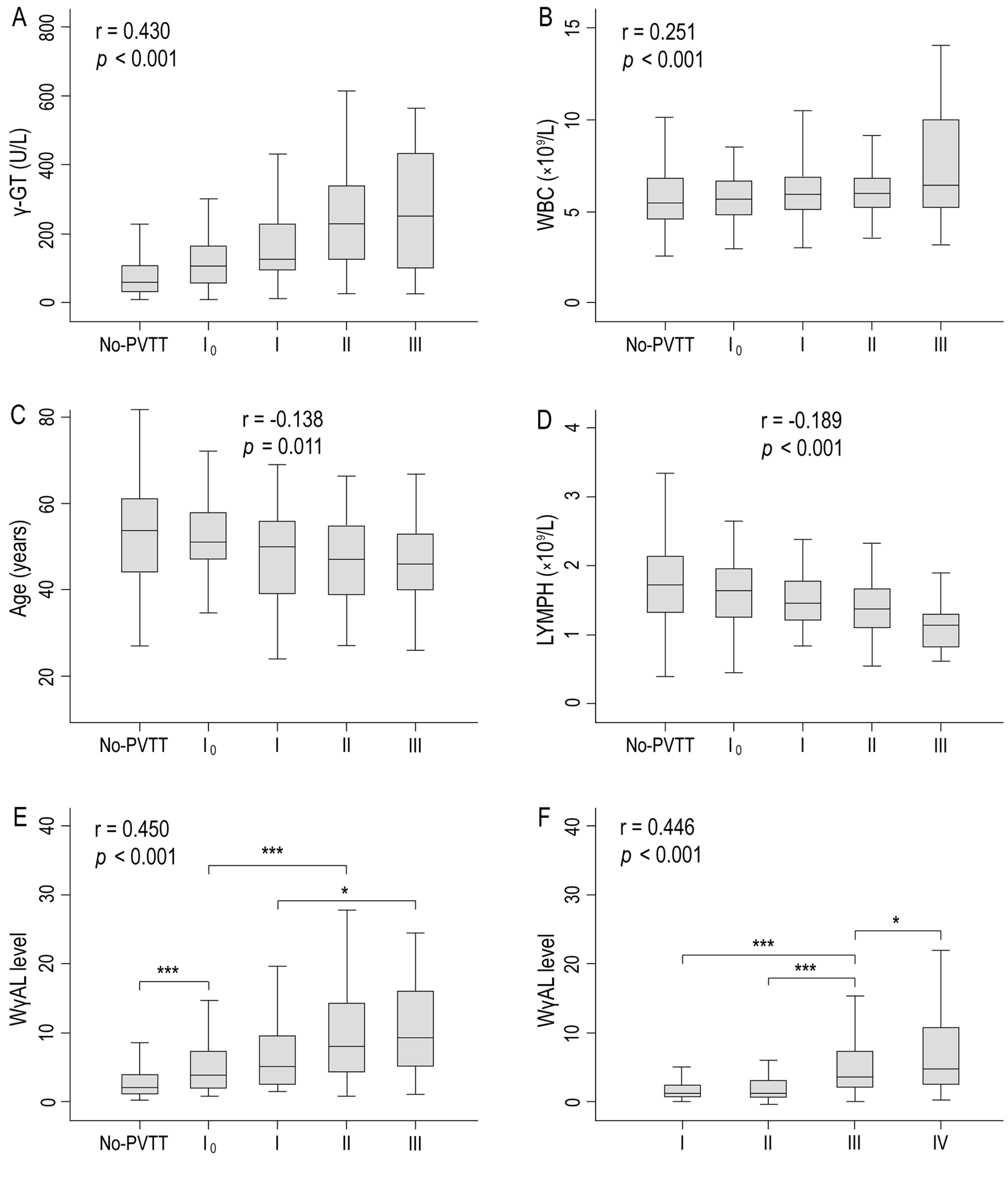

Supplementary Figure 1: Box plots of **(A)** serum γ-GT level, **(B)** WBC count, **(C)** age, **(D)** LYMPH count, and **(E)** WγAL index level according to the PVTT type, and **(F)** WγAL index level in relation to TNM stage in the validation cohorts (*, $P < 0.05$; **, $P < 0.01$; ***, $P < 0.001$).
